# Supplementary material for: Collaborative intelligence in AI: Evaluating the performance of a council of AIs on the USMLE
Source: PLOS Digit Health. 2025 Oct 9;4(10):e0000787. doi: 10.1371/journal.pdig.0000787 (PMC12510544; doi:10.1371/journal.pdig.0000787)
Supplement: S2 File — (ZIP) [file pdig.0000787.s002.zip › S2 Data Raw data files containing unprocessed question inputs and LLM outputs including transcripts of deliberations/STEP3_Question-18-1_Type-MC-NJ.docx]

**Facilitator** **[Round 1]:** A 45-year-old man comes to the office for an initial appointment because he would like you to fill out and sign a legal document at the request of his attorney. The patient works as a limousine driver. He states that he is filing a lawsuit against the limousine company that employs him because he developed post-traumatic stress disorder following a motor vehicle collision. He appears irritable and tense as he provides his history. He relays his symptoms by reading them aloud from a written list. At this time, which of the following is the most appropriate approach toward confirming the underlying diagnosis of this patient? (A) Administer amobarbital and then interview the patient (B) Ask the patient to provide a narrative with detailed description of the incident and of his symptoms (C) Interview the patient under hypnosis (D) Interview the patient while paying close attention to his willingness to make eye contact (E) Tell the patient he is exhibiting behaviors that are suggestive of malingering and see how he responds

**AI_C:** RESTATING THE QUESTION: The question essentially presents a suspected case of post-traumatic stress disorder (PTSD) of a 45-year-old limousine driver who claims to have developed the disorder following a car crash. The man is asking for a legal document to support his lawsuit against his employer. The question is asking what would be the most appropriate clinical approach to verify the accuracy of his claim. PLANNING: Assuming the patient truly has PTSD is one aspect. However, this could also be a case of malingering, where a person pretends to have an illness or takes on the role of a sick person for some form of secondary gain. This is often seen in legal or compensation situations. The man’s symptoms are read from a list, which may suggest that he's not genuinely experiencing them. To discern the true nature of the situation, we should prioritize an approach that allows us to objectively evaluate his claim. CLINICAL REASONING: Amobarbital administration and hypnosis are diagnostic tools for different scenarios and not typical in outpatient PTSD evaluation. Similarly, attention to eye contact is often insufficient to make a definitive diagnosis. Telling the patient that his behavior suggests malingering could induce hostility. Thus, the best approach likely involves a comprehensive and detailed discussion of the incident and its resulting symptoms. CONCLUDING: By asking the patient to provide a detailed description of the incident, we can gain insight into his emotional reactions and feelings both during and after the event. Such narratives are often marked by clear emotional responses in those truly suffering from PTSD. Furthermore, talking through his symptoms in detail may help characterize their intensity, frequency, and impact on daily life. RESPONSE: Hence, the most appropriate approach towards confirming the underlying diagnosis of this patient will be (B) Ask the patient to provide a narrative with detailed description of the incident and of his symptoms. This will help determine if he indeed meets the criteria for PTSD or suggests malingering.

**AI_B:** RESTATING THE QUESTION: This scenario involves a 45-year-old limousine driver who has requested a signed legal document to support his lawsuit against his employer, alleging that he developed post-traumatic stress disorder (PTSD) following a motor vehicle collision. He exhibits irritability and tension during the conversation. The question requires the most appropriate clinical approach to validate his PTSD diagnosis. PLANNING: Major assumptions in this case are: the patient is telling the truth and has PTSD; the patient is malingering; the patient's physical responses or cognitive state may help clarify his condition; or the specifics about his trauma and how he responds to recollection could shed light on his condition. It's necessary to evaluate each assumption critically and decide clinically which needs prioritizing. CLINICAL REASONING: Malingering is a possibility given the active lawsuit, but accusing the patient abruptly may jeopardize the clinician-patient relationship and halt productive communication. Thus, options involving risky interventions like administration of amobarbital or hypnosis, or challenging the patient's truthfulness directly, are not preferred. PTSD is a diagnosis made clinically through patient history and not based on eye contact. Revisiting the traumatic event in detail and understanding the patient's reactions could provide a better understanding of the psychological impact and point towards a PTSD diagnosis. CONCLUDING: Given the complexities, a sensitive approach would be to encourage the patient to relay his experience surrounding the traumatic event and his symptoms in detail. This would provide a better understanding of whether his symptoms align with the criteria for PTSD without causing unnecessary distress or conflict. RESPONSE: Thus, the most appropriate approach towards confirming the underlying diagnosis of this patient would be to ask him to provide a narrative with detailed description of the accident and his symptoms (Option B).

**AI_D:** RESTATING THE QUESTION: The question is asking which approach will be most effective in verifying whether the 45-year old limousine driver in question truly has post-traumatic stress disorder (PTSD). PTSD is a psychiatric condition that surfaces following events that entail significant trauma and is characterized by intrusive thoughts, avoidance behaviors, and hyper-arousal. PLANNING: There are multiple assumptions in this case. The primary assumption is that the patient has PTSD because he claims he developed it after a motor vehicle accident. Another assumption is that his irritability and reading of symptoms from a list might be indicative of either genuine PTSD symptoms or possible malingering to gain advantage in his lawsuit. It is important to note that PTSD diagnosis entails observing certain symptoms over a specific duration, in line with the DSM-5 criteria. In the process, we assume that certain methods like administering amobarbital, using hypnosis, analyzing eye contact, or outrightly suggesting malingering could aid confirmation. CLINICAL REASONING: Establishing PTSD entails detailed exploration of a patient's lived experiences and the symptoms manifesting over time. Presence of hyperarousal symptoms such as irritability, which overlaps with the description of our patient, is integral to diagnosing PTSD. While amobarbital and hypnosis could help uncover repressed memories, they are not standard methods for diagnosing PTSD. Eye contact can provide insights into a patient's non-verbal communication but it is not specific for PTSD. Suggesting malingering without substantial evidence would likely jeopardize the doctor-patient relationship. Asking for a detailed narrative would better conform to practices in psychological evaluation. CONCLUDING: From a clinical perspective, the most suitable method to diagnosing PTSD isn't a single symptom or an unconventional method like hypnosis, but a comprehensive evaluation of the patient's experiences and symptoms over time. This gives an opportunity for accuracy and reduces the likelihood of wrongly accusing a patient of malingering or overlooking real distress. RESPONSE: Therefore, the best way to approach confirming the underlying diagnosis of this patient's would be to "Ask the patient to provide a narrative with detailed description of the incident and of his symptoms" (Option B). The clinical diagnosis of PTSD requires the presence of symptoms that persist for more than one month following exposure to a traumatic event. A detailed narrative will help in assessing the symptomatology of the patient and thus guide in the diagnosis.

**AI_E:** RESTATING THE QUESTION: The question asked here revolves around a 45-year-old limousine driver who is seeking to have a legal document filled out and signed. The individual claims that after a car accident, he suffers from post-traumatic stress disorder (PTSD) and he wants to file a lawsuit against his employer. The question wants us to decide the best possible approach to confirm his diagnosis of PTSD. Potential strategies range from the use of a barbiturate (amobarbital), obtaining a detailed narrative of the incident and symptoms, hypnotic interview, interview focusing on eye contact, or suggesting the presence of malingering behaviors. PLANNING: In order to confirm this patient's diagnosis, we need to explore all alternatives. This involves examining our assumptions about the patient's story, his symptoms, and his behavior. One assumption is that the patient is telling the truth about his trauma and subsequent symptoms of PTSD. The other is that the patient might be malingering, thus exaggerating or feigning his symptoms for personal gain, in this case, likely the lawsuit. In distinguishing between these assumptions, it is crucial to prioritize clinical approach that would allow comprehensive and objective assessment of patient's psychopathology while guarding against potential manipulation. CLINICAL REASONING: For clinical scenario such as this, it's vital to follow standard procedure of diagnosing mental conditions. This usually involves exploring the patient's story in depth, obtaining specifics about the traumatic event, identifying the symptoms, and evaluating their impact on the individual's life. Assessing the sincerity of the narrative and looking for consistencies or inconsistencies can provide important insight. Techniques such as hypnosis or the use of amobarbital (a medication that can encourage the disclosure of information through relaxation and lowering of inhibitions) are inconsistent with standard psychiatric interviews and might impair patient's ability to give informed consent. Explicitly mentioning malingering tends to create an adversarial relationship between patient and physician, which impairs further assessment and may not be productive in all settings. Monitoring eye contact might help in understanding patient's level of comfort during interview but it is not specific to any particular diagnosis. CONCLUDING: Based on this thought process, the best approach to confirming the diagnosis in this situation should focus on conducting a proper, comprehensive, and structured clinical interview about the traumatic event and its subsequent symptoms. This approach allows professional judgement on the sincerity, consistency, and severity of symptoms, which are needed to determine if there really is a PTSD or potential malingering. RESPONSE: Therefore, the most appropriate approach toward confirming the underlying diagnosis of this patient is (B) Ask the patient to provide a narrative with a detailed description of the incident and of his symptoms. This approach is aligned with the standard clinical practice of diagnosing PTSD, which involves a deep dive into the patient’s symptoms, extent of impairment, and other potential clinical features of the disorder.

**AI_A:** (1) RESTATING THE QUESTION: The question is asking what's the optimal method to ascertain the underlying diagnosis of a limousine driver in his mid-forties who is seeking a health certificate for a lawsuit against his employer. He alleges suffering from PTSD after a car accident. The symptoms are relayed via a recital from a prepared list, and the man seems tense and irritable. Our options involve use of different clinical approach techniques including amobarbital induction, narrative description of the incident and symptoms, hypnotic interview, close attention to his eye contact, or suggesting the possibility of malingering. (2) PLANNING: In evaluating this scenario, we need to keep in mind several considerations. The patient's tension and irritability could be manifestations of distress from PTSD, or could indicate defensive behavior if he's overstating or making up symptoms -- malingering. Though he's handed over a list of symptoms, it doesn't clarify the severity, frequency, or extent to which they're impacting his daily life. With these factors, the assumptions to be tested are: (i) he indeed has PTSD, (ii) the PTSD was indeed caused by the motor vehicle accident, and (iii) whether malingering is present or not. (3) CLINICAL REASONING: To narrow down these assumptions, we must apply principles of psychopathology, clinical examination, and ethical boundary setting. To diagnose PTSD, it's necessary to collect personal narratives about the trauma experience, physiological responses, recurrent unwanted and distressing memories, and changes in emotional and behavioral responses. Amobarbital interview or hypnosis aren't standard methods for ascertaining PTSD as they impose a risk of creating false memories or exposing vulnerabilities. Evaluating eye contact alone cannot confirm or rule out PTSD either. Suggesting malingering from the outset is premature, potentially offensive and can damage the clinician-patient rapport. Therefore, option B for thorough exploration of the incident and the personal impact of symptoms stands out as the most ethical, scientifically valid, and comprehensive approach. (4) CONCLUDING: A comprehensive investigation of the traumatic incident and its psychological outcomes as per option B is clinically appropriate. It allows us to evaluate the diagnosis of PTSD as per its defined criteria. This method allows for better understanding of the patient's experience, emotional responses, symptom severity, and specific ways the event has disrupted his life. (5) RESPONSE: The most appropriate approach to confirm the underlying diagnosis of this patient would be to ask him to provide a detailed narrative of the incident and his symptoms (B). This approach allows us to analyze the probabilities our assumptions, evaluate the clinical picture, and arrive at a diagnosis that best aligns with the evidence provided by the patient's account.

**Facilitator** The consensus among the AI responses is that the most appropriate approach to confirm the underlying diagnosis of the 45-year-old limousine driver, who alleges suffering from PTSD after a car accident, is to ask him to provide a detailed narrative of the incident and his symptoms (Option B). This approach is in line with standard clinical practice for diagnosing PTSD and allows for a comprehensive evaluation of the patient's experiences, emotional responses, symptom severity, and the specific ways the event has disrupted his life. The AI responses also agree that other options such as administering amobarbital, conducting a hypnotic interview, focusing on eye contact, or suggesting malingering are not preferred. These methods are either not standard for diagnosing PTSD, risk creating false memories or exposing vulnerabilities, or could potentially damage the clinician-patient rapport. The AI responses highlight the importance of considering the possibility of malingering given the active lawsuit, but caution against accusing the patient without substantial evidence. They emphasize the need for a sensitive and ethical approach that encourages the patient to relay his experience without causing unnecessary distress or conflict. In conclusion, a detailed narrative of the incident and symptoms is the most clinically appropriate, scientifically valid, and comprehensive approach to confirm the underlying diagnosis of this patient.
